# Ratio-Based Analysis of Differential mRNA Processing and Expression of a Polyadenylation Factor Mutant *pcfs4* Using Arabidopsis Tiling Microarray

**DOI:** 10.1371/journal.pone.0014719

**Published:** 2011-02-25

**Authors:** Jianti Zheng, Denghui Xing, Xiaohui Wu, Yingjia Shen, Diana M. Kroll, Guoli Ji, Qingshun Quinn Li

**Affiliations:** 1 Department of Automation, Xiamen University, Xiamen, Fujian, China; 2 Department of Botany, Miami University, Oxford, Ohio, United States of America; Memorial Sloan-Kettering Cancer Center, United States of America

## Abstract

**Background:**

Alternative polyadenylation as a mechanism in gene expression regulation has been widely recognized in recent years. Arabidopsis polyadenylation factor PCFS4 was shown to function in leaf development and in flowering time control. The function of PCFS4 in controlling flowering time was correlated with the alternative polyadenylation of *FCA*, a flowering time regulator. However, genetic evidence suggested additional targets of PCFS4 that may mediate its function in both flowering time and leaf development.

**Methodology/Principal Findings:**

To identify further targets, we investigated the whole transcriptome of a *PCFS4* mutant using Affymetrix Arabidopsis genomic tiling 1.0R array and developed a data analysis pipeline, termed RADPRE (Ratio-based Analysis of Differential mRNA Processing and Expression). In RADPRE, ratios of normalized probe intensities between wild type Columbia and a *pcfs4* mutant were first generated. By doing so, one of the major problems of tiling array data—variations caused by differential probe affinity—was significantly alleviated. With the probe ratios as inputs, a hierarchy of statistical tests was carried out to identify differentially processed genes (DPG) and differentially expressed genes (DEG). The false discovery rate (FDR) of this analysis was estimated by using the balanced random combinations of Col/*pcfs4* and *pcfs4*/Col ratios as inputs. Gene Ontology (GO) analysis of the DPGs and DEGs revealed potential new roles of PCFS4 in stress responses besides flowering time regulation.

**Conclusion/Significance:**

We identified 68 DPGs and 114 DEGs with FDR at 1% and 2%, respectively. Most of the 68 DPGs were subjected to alternative polyadenylation, splicing or transcription initiation. Quantitative PCR analysis of a set of DPGs confirmed that most of these genes were truly differentially processed in *pcfs4* mutant plants. The enriched GO term “regulation of flower development” among PCFS4 targets further indicated the efficacy of the RADPRE pipeline. This simple but effective program is available upon request.

## Introduction

Polyadenylation is one of the essential processes during the maturation of most mRNAs in eukaryotic cells. Accumulating evidence suggests that this process has been widely employed by higher eukaryotes, through alternative polyadenylation (APA), to regulate gene expression and therefore plays roles in specific biological functions[1]–[13]. Arabidopsis polyadenylation factor PCFS4 has been shown to function in the alternative polyadenylation of *FCA* pre-mRNA and therefore affects the regulation of flowering time [13]. *FCA* encodes two major transcript isoforms, *FCA-gamma* and *FCA-beta*. *FCA-gamma* is derived from the regular poly(A) site (distal to the promoter) and encodes the functional FCA, while *FCA-beta* is from the poly(A) site within intron 3 (proximal to the promoter) and is non-functional [5], [14]. By physically interacting with other polyadenylation factors, PCFS4 promotes the proximal poly(A) site usage and thus controls the ratio of the two transcript isoforms of *FCA* and flowering time [13], [15]. In addition to flowering time, PCFS4 also functions in leaf development, as indicated by its mutant *pcfs4*-1 abnormality [13]. However, PCFS4's control of leaf morphology could not be explained by its role in APA of *FCA* since the leaf morphology was not affected in *fca* mutants [13]. Therefore, we reasoned that in addition to *FCA,* there may be other target(s) of PCFS4 that mediate its function in leaf development, likely through a molecular mechanism similar to the APA of *FCA*. To explore this possibility, we employed a whole genome tiling microarray technology to search the Arabidopsis transcriptome for the differentially processed genes (DPG, defined as those genes whose pre-mRNA processing was altered in the *pcfs4* mutant) and differentially expressed genes (DEG, defined as those genes whose steady-state level of mRNA abundance was altered, but the mRNA processing was not affected in the *pcfs4* mutant). The DPGs are likely the direct targets of PCFS4 and the DEGs may be the indirect or secondary targets.

Genomic tiling microarray technology has been used in a variety of applications in plants including empirical annotation of the transcriptome, Chip-chip studies, mapping the methylome, and identification of DNA polymorphism [16]–[26]. In the genomic tiling microarray, specifically in the oligonucleotide tiling array, the probes are designed to cover the whole genome with a very high resolution [27]. For example, Arabidopsis tiling 1.0R array (Affymetrix) tiles 6.4 million 25-mer probes per array with a resolution of about 35 bp [24]. High probe density results in each of the transcription units being represented by multiple probes, in contrast to “one-probe-one-gene” in expression microarrays. Multiple probes for a given transcription unit render abundant measurements which allows for further statistical evaluations, and therefore, leads to more accurate results. Furthermore, if a gene encodes multiple transcript isoforms, the gene could be revealed by plotting the probe signal intensities against the gene structure [28], [29]. It is these features that make it possible to use Arabidopsis tiling 1.0R array to study not only the DEGs, but also the DPGs in the *pcfs4* mutant.

The power of the whole genome tiling array is due to its high probe density, which results in high volume of data output. However, at the same time, the massive volume of data and the inherent nature of the data - low signal-to-noise ratio - make the extraction of biologically meaningful information challenging [27], [30]. In the last several years, a few analysis methods for tiling array data have been published, including TileMap, gSAM, Segmentation, BASIS, ARTADE and TileScope [28], [31]–[34]. The TileMap, Segmentation, ARTADE and TileScope methods focused on mapping deduced transcript units on the genome; gSAM was designed to find the differentially expressed transcription units, but specifically deals with the experimental design in a manner of time series; BASIS was designed to identify the differentially expressed splicing isoforms, but has the pre-requisite that the splicing isoforms have already been defined. In addition, different methods often deal with data from different tiling array platforms and/or for specific applications. Although one method might be adapted to analyze data from different platforms and/or for a different application, the adaptation is not always straightforward.

To identify the DPGs and DEGs of PCFS4, we used Arabidopsis tiling 1.0R array from Affymetrix and developed a data analysis pipeline, termed “Ratio-based Analysis of Differential mRNA Processing and Expression” (RADPRE). One of the major problems with tiling array data analysis is that the intensities of the multiple probes representing a given transcript unit (a transcript isoform) or annotation unit (such as an exon) are highly variable [27]. Heterogeneous probe affinity is one of the many factors contributing to the variation [35]. Efforts have been made to remove the probe affinity effects using both computational and empirical techniques, which have been especially helpful in mapping transcription activity [29], [35]–[37]. Given the specific purpose of this study and that the probe intensities between the two experimental conditions are highly correlated, we designed the RADPRE analysis pipeline, efficiently alleviating the problem of probe affinity variations by generating Control/Treatment (or Treatment/Control) ratios of probe intensities. The ratios representing a transcript or annotation unit were further subjected to a classical T-test and F-test (ANOVA) to determine whether a given gene was being differentially processed (DPGs) and/or expressed (DEGs). This protocol, although designed and used here for the identification of DPG and DEG targets of PCFS4, should be applicable to study the effects of other factors related to mRNA processing (splicing, polyadenylation, etc), transcription initiation, and/or gene expression.

## Results

### Data description

**Figure 1:**
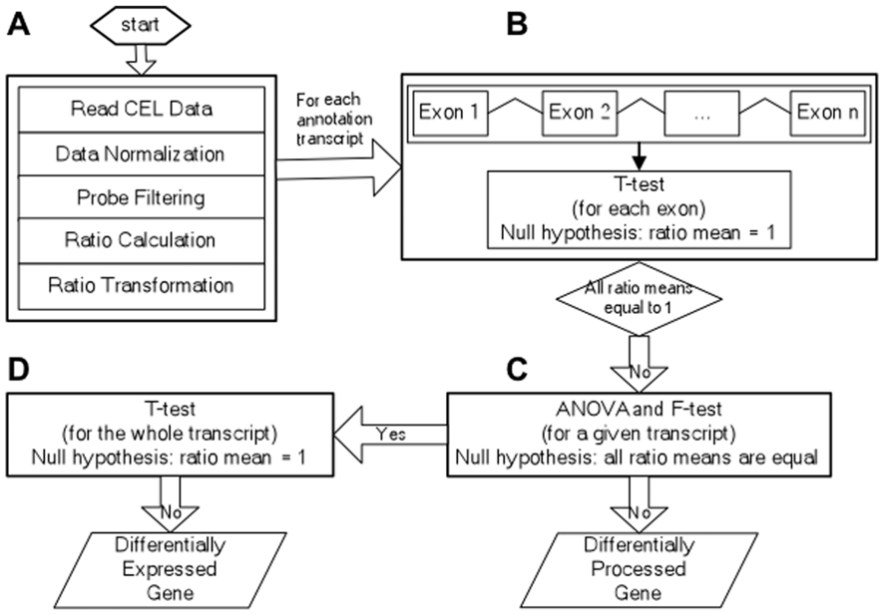
A flow-chart of RADPRE analysis pipeline. **A**) Preprocessing of data including background correction, across-array normalization, probe filtering and trimming, ratio generation, and log-transformation. **B**) To identify transcripts with at least one of its exon ratio means not equal to one, a one-sample two-tails T-test was applied to every exon of an annotated transcript with the null hypothesis that the ratio mean of the exon was equal to one. **C**) For those transcripts identified from the T-test in (B), a one-way ANOVA and F-test was performed for each transcript with its exons as the “level” parameter. Every transcript with the ratio means of all its exons being equal would be a putative DEG target. Otherwise, the transcript would be a direct DPG target. **D**) A further one-sample two-tails T-test was applied to every one of the putative DEG targets from (C) to test whether the ratio mean of the whole transcript was equal to one. If the ratio mean was not equal to one, the transcript would be a DEG target.

There were six CEL file data (wt1, wt2, wt3, pcfs4.1, pcfs4.2 and pcfs4.3) from the hybridization of Arabidopsis tiling 1.0R array with the targets prepared from three biological replicates of each wild type Col (WT) and the *PCFS4* mutant (*pcfs4-1*) grown in a randomized block design. The suffix number of the file name denotes the block number. The data was analyzed according to the steps shown in Figure 1. The underlying principle, the analysis details and the results of each step are described as follows.

### Preprocessing of the raw data

For a given probe, its signal intensity recorded in the CEL file could be attributed to the relative abundance of its corresponding target in the sample. Other factors include the background (defined as the signal intensity of a probe generated when its corresponding target was missing from the population), the spatially uneven hybridization on the same array chip, the unequal amount of targets applied to different array chips and the heterogeneous probe affinities [35], [38]. The effects of the first three factors were removed through background correction and across array normalization. The probe affinity effect was removed by generating the ratio of WT/*pcfs4* as detailed below.

### Background correction and across-array normalization

**Figure 2:**
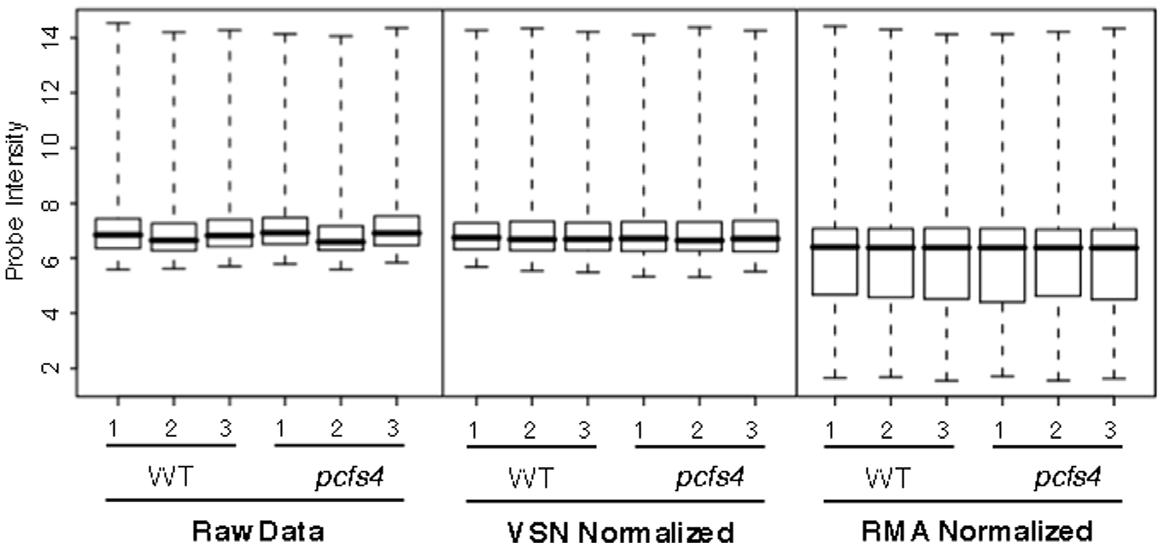
Box plots of log-transformed raw data (left panel), VSN-normalized (middle panel) and RMA-normalized (right panel) data. The across-array variations were significantly reduced after normalization.

The quality of the array data is essential for extracting biologically meaningful information. Therefore, the six CEL file data were first checked for their quality through “exploratory” data analysis with raw and log-transformed intensities [39]. The even distributions of signal intensities across the chips indicated high quality original data (Figure S1). Among the available methods for background correction and across-array normalization, “VSN” and “RMA” were chosen for the processing of our array data [38], [40]. The box-plots of before- and after-normalization data clearly indicated that the normalization removed the across-array bias and rendered the data distribution more consistent among the 6 data files (Figure 2). Although there was no significant difference observed between VSN and RMA normalizations, the signal intensities were less varied with the former method (Figure 2). Therefore, the down-stream data analysis was carried out using the “VSN” normalized data.

### Ratio generation

**Figure 3:**
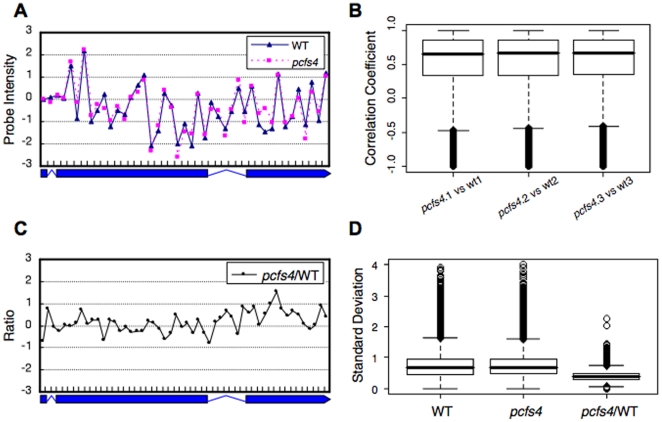
The variation of probe intensity across the same annotation unit was significantly alleviated by generating a ratio between WT and the *pcfs4* mutant. **A**) A plot of the probe intensities normalized against the average of all the probes across the annotation unit. The probe intensities fluctuated dramatically among probes, yet were highly correlated between wild type (WT) and mutant (*pcfs4*). **B**) The correlation coefficients of all exons with more than three probes were first calculated and then box-plotted. The median correlation coefficient was around 0.67 with the majority between 0.34 and 0.86. **C**) A plot of probe intensity ratios across the annotation unit. With the ratios, the variation among the probes was significantly reduced compared to the variation of intensities in (A). **D**) The variations of probe intensities or ratios across every exon with more than three probes were measured with standard deviation and then box-plotted. With ratios (*pcfs4*/WT), the median Standard Deviation (Std) was around 0.40, in contrast to the median Std of probe intensities of WT or *pcfs4*, 0.67 and 0.69.

**Figure 4:**
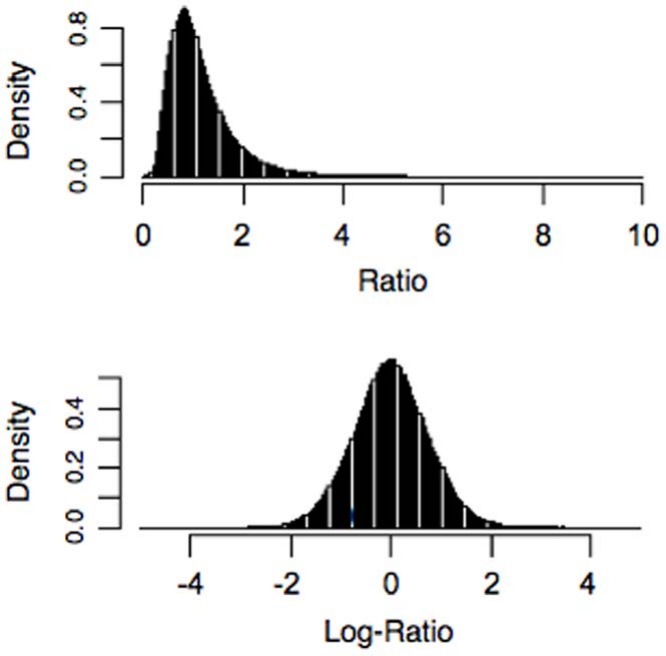
The density distribution of ratios (upper-panel) or log-transformed ratios (lower-panel). The latter was largely normal.

As in other tiling array data, the probe intensities of the data, which were supposed to represent the same transcript unit, were highly varied due to the heterogeneous probe affinity (Figure 3A) [27]. Despite this, the probe intensities seemed highly correlated across multiple samples (among replicates and/or treatments) (Figure 3A, B). Indeed, the box plot of correlation coefficients from exons with ≥3 probes revealed that 50% of the correlation coefficients ranged from 0.34 to 0.86, with a median of 0.67 between the WT and the *pcfs4* mutant (Figure 3B). Therefore, the probe affinity effects might be removed by generating the ratio of WT to *pcfs4* as previously suggested [27]. As shown (Figure 3C), the variation of the ratios representing the same annotation unit was dramatically reduced in comparison to those of the same set of probe intensities for either wild type or mutant (Figure 3A). The variations measured with the standard deviation for the probe intensities and ratios for all exons (≥3 probes) were box-plotted. Based on the box-plot, the variation among the ratios was significantly lower than the probe intensities of either the WT or *pcfs4* mutant (Figure 3D). Thus, the down-stream statistical analyses were performed with the probe intensity ratios instead of the probe intensity per se. Since the distribution of the probe intensity ratios skewed to lower values, the ratios were first log-transformed. As shown in Figure 4, the distribution of log-transformed (base 2) ratios was largely normal (Table S1), which was required for the down-stream statistical analyses [41].

### Construction of CDF files

Affymetrix provides the component definition file (CDF) “At35b_MR_v04-2_TIGRv5.bpmap” for the Arabidopsis tiling 1.0R array, in which the detailed genomic information of the probes were denoted. For the purpose of this study, a new CDF file, “AtTA.cdf”, was constructed based on the following criteria: 1) only the perfect match (PM) probes with a unique match position in the genome were considered; 2) the probes spanning the borders of annotation units were removed; and 3) the annotation units represented by a single probe were not considered. The reasons for trimming the probes and annotation units are as follows. The mismatch probes (MM) in the tilling array were intended to be references for the non-specific hybridization to PM probes. However, studies indicated that although MM might help to remove the non-specific signal intensity to some extent, as many as 30% of MM probes had intensities higher than their corresponding PM probes, thus defeating the purpose of using the MM intensity to adjust the PM intensity [27], [38]. For the probes spanning the border of the annotation units, their corresponding targets only partially matched the probes, rendering those probes equivalent to the non-perfect matches. Thus, the signal intensities of these probes were underestimated for the abundance of their corresponding targets. For those annotation units represented by a single probe, only 3 ratios (for each probe) were available for the down-stream calculation of T-statistics and F-statistics. The sample size was likely too small to utilize these statistical tests. Therefore, those annotation units were not considered for the analysis. Depending on the analysis purpose, the probes were either grouped based on annotation units (such as exons) or transcription units (transcript isoforms).

### Statistic tests to identify DPGs and DEGs

**Figure 5:**
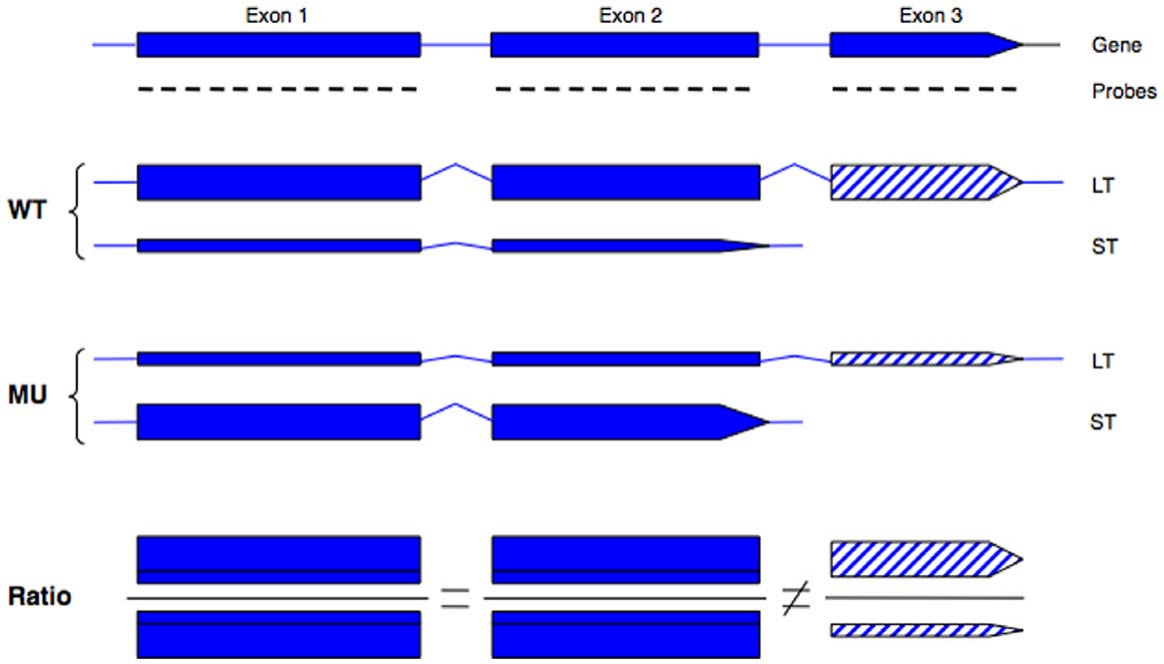
Schematics of how a DPG gene could be identified based on the ratios of its exons between wild type Col (WT) and the *pcfs4* mutant (MU). The gene structure was shown on the top of the graph with filled boxes denoting exons, lines denoting introns, and the short lines under the exons denoting the tiling array probes. The gene could generate two transcripts, a long (LT) and a short (ST), with LT derived from a distal poly(A) site and containing exon 3 (hatched box), and ST from a proximal poly(A) site within intron 2. The thickness of the box represents the relative abundance of the transcripts. The relative abundance of two transcripts was altered between WT and MU due to the shift of the poly(A) site usage between WT and MU. The measured abundance of each exon was based on its corresponding probes, which reflected the sum of two transcripts. The measured abundance of exon 1 and 2 was the same between WT and MU, but that of exon 3 was different. Therefore, the ratio of exon 1 and 2 between WT and MU was equal to 1, but different from the ratio of exon 3. In that case, the poly(A) site choice was not affected by the mutant and the relative abundance of the two transcripts would be the same between WT and MU. The ratio of the measured abundance between WT and MU would be equal for all three exons.

The underlying concept to identify the DPG targets of PCFS4 is depicted in Figure 5. With a given unknown gene, it was assumed the gene had two major transcripts derived from its pre-mRNA by APA, as in the case of *FCA* [13]. For example, if the choice of poly(A) sites is controlled by PCFS4, then the ratio of exon 3 abundance between WT and *pcfs4* would be altered such that the ratio of exon 3 would deviate from 1 (more or less than 1) while the ratios of exon 1 and exon 2 should be equal to 1 (Figure 5). This case could be generalized as follows: if the APA of a given gene is controlled by PCFS4, then 1) the WT/mutant ratio of one or more exons would not be equal to one, and 2) the ratio of at least one exon would not be equal to the ratios of other exons. This generalization would also hold true if a gene's splicing and/or transcription initiation was affected by PCFS4. In the case of a given gene being a DEG target, which was defined as whole gene expression difference rather than an individual exon difference, the ratios between WT and *pcfs4* of all exons for this gene would have the same degree of deviation from 1 (if larger than 1, under expressed in *pcfs4*; if smaller than 1, over expressed in *pcfs4*).

Based on the concept described above, hierarchical statistical analyses were applied to the log-transformed ratio data to identify both DPG and DEG targets of PCFS4 (Figure 1B, C, D). A student one sample two-tails T-test was first performed on all the exons of a given transcript of a given gene. The null hypothesis was the mean of log-transformed ratios for the exon was equal to zero (Figure 1B). If all mean ratios of every exon within the transcript were equal to one (indicative of no change between WT and the mutant), the gene was not a target of PCFS4. Otherwise, a one-way ANOVA followed by F-statistic were conducted to test whether the ratio means of each and every exon were equal (Figure 1C). If the answer is “yes”, then the corresponding gene was considered to be a candidate DEG target of PCFS4 (indicative of uniformly changed expression levels of the whole transcript). Otherwise, the gene was considered to be a DPG target of PCFS4 (indicative of partial changes of the transcript; Figure 1C). To increase the specificity for the candidate DEG targets of PCFS4, a further T-test was carried out with the null hypothesis being the ratio mean of all probes within the entire transcript was equal to one. If the null hypothesis was accepted, then the gene was not a DEG target of PCFS4. Otherwise, the gene was a target of PCFS4 (Figure 1D).

To estimate the false discovery rate (FDR) of the above analyses, we applied the concept of using balanced random combinations of samples from two conditions. Specifically, each balanced random sample consisted of two WT/*pcfs4* and two *pcfs4*/WT ratios [23], [42]. Since we had only three replicates of each WT and *pcfs4*, a fourth sample was generated by taking the geometric average of the three replicates for WT or *pcfs4* so that the random combinations would be balanced (meaning two WT/*pcfs4* ratios and two *pcfs4*/WT ratios for each combination). With the RADPRE analysis of each random combination, a certain number of genes would be called significant based on a certain p-value (Table 1). The average number of significant genes from all possible combinations served as the number of falsely discovered genes. FDR was calculated based on the number of false discovered genes and the number of identified DPG or DEG targets of PCFS4.

**Table 1:** The false discovery rate (FDR) of Differentially Processed Genes (DPGs) and Differentially Expressed Genes (DEGs).

	P-Value			
	T-test	F-test	# of genes	FDR (%)
DPGs	0.05	0.05	1013	7.3
	0.01	0.05	515	3.0
	0.01	0.01	176	2.8
	0.001	0.05	142	1.9
	0.0001	0.05	68	1.0
DEGs	0.05	0.05	3546	14.8
	0.01	0.05	1554	7.9
	0.001	0.05	485	5.0
	0.0001	0.05	220	3.5
	0.00001	0.05	114	1.8

Note: T-test and F-test, as depicted in Figure 1b and c; the p-value for the T-test on the whole transcript level (Figure 1d) was set at 0.05 (see Results section).

**Figure 6:**
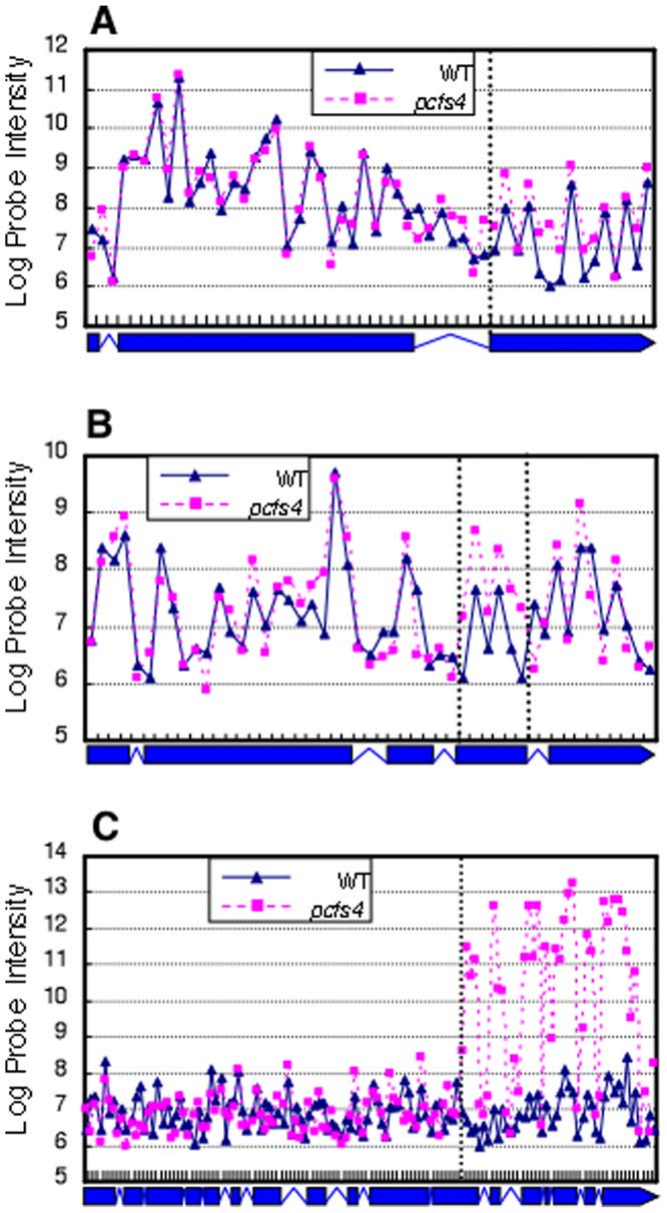
The DPGs of PCFS4 could be explained by 3 simple models: alternative polyadenylation, alternative splicing and alternative transcription initiation, as shown by A) At4g38160.2, B) At5g46490.2, and C) At5g52910.1, respectively. Each data point represented the average of three replicates. The dotted vertical lines separate annotation units of the gene with or without difference between wild type (WT) and *pcfs4* mutant. The corresponding gene structure was presented under each plot with boxes denoting the exons and lines denoting the introns.

Following the RADPRE analysis, 68 DPG and 114 DEG targets of PCFS4 were identified with the FDR of 1% and 2%, respectively (Table 1; Table S2 and Table S3). Close examination of the probe intensities along the gene bodies revealed that the 68 DPG targets could be explained by 3 simple models: 1) alternative polyadenylation, 2) alternative splicing, and 3) alternative transcription initiation (Table S2). An example of each model is illustrated in Figure 6. The examples shown here are also supported by data from other sources. Both genes, At4g38160 (Figure 6A) and At5g46490 (Figure 6B), have more than one gene model and supported by more than one full-length cDNAs from The Arabidopsis Information Resources (TAIR; www.arabidopsis.org). These transcripts are the results of both alternative polyadenylation and alternative splicing. In the case of At5g52910 (Figure 6C), only one full-length cDNA supported the 3'part of the gene model, there are, yet, numerous of ESTs supporting the expression of the 5'part of At5g52910 (TAIR).

It is noteworthy that categorizing each gene into one of the models was purely based on the probe intensities. Given the actual transcript isoforms are unknown for each gene (tiling array does not offer individual sequence information), this classification is essentially tentative and by no means should be used to replace further experimental exploration.

### Verification of DPG targets of PCFS4

**Figure 7:**
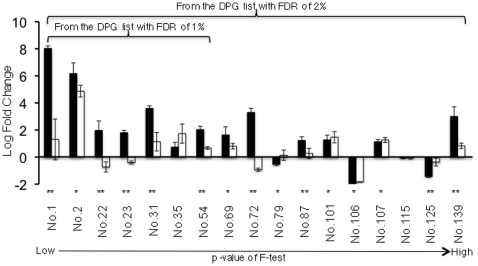
Quantitative PCR confirmation of the DPG targets of PCFS4. The average log fold change of *pcfs4*/WT is shown for two parts of each gene with a capped vertical line representing the standard error. The filled and open boxes represent the two parts of each gene being tested. The gene part showing a larger difference between wild type and mutant based on the tiling array data is represented by the filled box while the part showing less (or no) difference is represented by the open box. The double asterisk “**” denotes the genes showing significant difference (p = 0.05) for the ratios of two parts of each gene by qPCR. The single asterisk “*” denotes the genes whose one or both parts showed significant difference (p = 0.05) between wild type and the *pcfs4* mutant, but whose ratio of two parts showed no significant difference. The first 7 genes were from the DPG list with the FDR of 1% and they were a part of the total 17 genes from the DPG list with the FDR of 2%.

To verify that the DPG genes were truly differentially processed, a set of DPG targets chosen to cover the whole range of p-value (from low to high) were tested using quantitative (real-time) PCR. Originally, we chose the first two genes of every 10 DPGs to make the sample list. Due to technical problems such as 1) two or more gene models correspond to the same gene, 2) no suitable primers could be designed for a given gene, or 3) the failed qPCR, the sample list ended up with a total of 17 genes. Seven of these genes were from DPGs with 1% FDR, and 10 from genes between 1% to 2% FDR (Figure 7). For each gene, two pairs of primers were designed. One pair targeted on the exon(s) whose expression showed no (or less) difference between WT and *pcfs4* while the other pair targeted on the exon(s) that was differentially expressed (or showed a larger difference). The first strand cDNA samples were synthesized from the same RNA samples prepared for the tiling array hybridization targets.

For the 7 genes tested from the DPG list (68 genes) with the FDR of 1% (Table S2), 5 of them showed significant ratio differences between two parts of each gene (Figure 7). Of the two genes showing no significant ratio differences, gene No. 2 showed an expression difference between wild type and *pcfs4* mutant, while gene No. 35 has a p-value (0.08) close to the critical p-value of 0.05. Thus, FDR of 1% is a reasonable estimate of the “true” false discovery rate of the top 68 DPGs.

For FDR at the 2% level, a total of 17 genes were tested, 9 of them showed significant ratio differences (Figure 7). For the rest 8 genes, 6 of them showed differences between wild type and mutant for at least one part of each gene, suggesting these genes were potential DEG targets. These results indicated that the FDR of 2% for the 142 DPGs was an under-estimate of “true” false discovery rate. However, most of the falsely discovered genes were DEG targets of PCFS4. For the DPGs being tested with qPCR, both the changing direction and scale are consistent between qPCR and the tiling array results for most of them. The only exceptions are genes No. 35, No. 101 and No. 107, which are not the DPG targets of PCFS4 (Figure 7 and data not shown).

In summary, when the p-value of F-test was set low, the estimated FDR of DPGs was reasonably accurate and most of the DPGs were genuinely differentially processed in the *pcfs4* mutant. With the increase of p-value, the estimated FDR became less accurate and more genes than estimated were not “truly” differentially processed.

### Gene Ontology (GO) analysis of the DPG and DEG targets of PCFS4

To examine what biological processes the DPGs of PCFS4 may be involved in, a GO analysis of the 68 DPGs was performed using GOEAST [43]. Results showed that “response to stress” and “regulation of flower development” were among the most enriched GO terms with p-values less than 0.0001 (Table S4). The GO term “regulation of flower development” was significantly enriched in the 68 DPG targets, which further strengthens the efficacy of RADPRE in the identification of authentic DPG targets since the major phenotype of *pcfs4* is delayed flowering time [13]. While the enrichment of the genes involved in “response to stress” was unpredicted, it suggested that PCFS4 very likely played a role in stress responses. Furthermore, it has already been documented that stress could cause changed flowering time [44]–[47]. Thus, it is possible that the altered processing of the genes involved in stress response contributed, to some extent, to the flowering time delay in the *pcfs4* mutant.

Given that the genes involved in “regulation of flower development” and “response to stress” were enriched in the DPGs, the direct targets of PCFS4, the two GO terms might also be enriched in all targets (direct and indirect) of PCFS4. The GO analysis of the combined DEGs and DPGs using GOEAST proved this reasoning (Table S5). Both GO terms, “regulation of flower development” and “response to stress,” were indeed enriched in the pooled targets (Table S5). In addition, “sulfur metabolic process,” “circadian rhythm” and “S-glycoside metabolic process” were among the most enriched GO terms. It is well known that circadian rhythm is tightly related to flowering time and the genes in the two terms were also overlapped to some extent (Table S5) [48]–[50]. Whether the “sulfur metabolic process” and “S-glycoside metabolic process” were also related to flower development and/or stress response needs to be further explored. Alternatively, PCFS4 could function directly in these two processes.

## Discussion

To identify additional targets of PCFS4, we performed a set of tiling array experiments and developed an analysis pipeline, RADPRE, for Affymetrix Arabidopsis tiling array data. Using this pipeline, we identified some potential direct and indirect targets. The validity of these targets will be subjected to further testing and confirmations. One of major problems for tiling array data is the probe intensity fluctuation within a gene or a gene transcript due to the heterogeneity of probe affinities to their matching sequences [27]. For studies examining the differences between biological samples from two experimental conditions, the probe affinity effects may be alleviated by using the ratios of probe intensities of one sample to the other [27]. Indeed, the probe intensities among the replicates (within WT or *pcfs4* mutant) or between treatments (WT and *pcfs4*) were highly correlated (Figure 3). By generating the ratios between WT and *pcfs4* mutant, variation across probes within a transcription unit was significantly alleviated (Figure 3). With the log-transformed ratios, which largely followed the Gaussian distribution (Figure 4), hierarchical statistical tests were performed in RADPRE for the annotated transcript isoforms of each gene to find the DPG or DEG of PCFS4 (Figure 1).

In this study, RADPRE was used to process data from a randomized block design for two biological conditions. The ratio generation was straight-forward since the wild type replicate was simply paired with the replicate of mutant within the same block (wt1/pcfs4.1; wt2/pcfs4.2 and wt3/pcfs4.3). However, if the experiment was carried out with a completely randomized design, the ratio generation could have been complicated since the pairing method for the wild type and mutant replicates would not be explicit. One solution would be to randomly pair the replicates of wild type and mutant. In this case, there would be nine combinations, resulting in nine ratios for each probe. Since the nine ratios would not be totally independent of each other, the degrees of freedom for the down-stream statistical analysis would be difficult to define. A simple solution would be to choose three independent ratios to represent the probes. This way, if there was no block effect, it would be equivalent to the randomized block design. Otherwise, the power of statistic justification might be compromised by the block effect. Therefore, as with other pipelines, appropriate experimental design for RADPRE is important and the statistical parameters may need to be adjusted accordingly.

Since RADPRE analysis could differentiate the DPG from the DEG targets, it could also be applied to experiments that simply look for differentially expressed genes. This means Arabidopsis tilling 1.0R array could be used as an expression array by this pipeline. A recently published method was specifically dedicated to this purpose [17]. Our method, however, can be used to extract both DPG and DEG information. The differentially processed genes are of special interest not only for investigating the roles of polyadenylation factors, but also for the splicing and transcription factors that may have an impact on the transcript compositions or expression levels. Given that RADPRE analysis was based on the annotated structures of genes, it has to rely on accurately annotated genomes. RADPRE is a fairly good choice for its simplicity and specificity with an estimated FDR of 1%. This FDR closely reflects the true false discovery rate since 6 of 7 DPG targets were verified with real-time PCR analysis (Figure 7). However, caution needs to be taken for using the FDR when the DPG list is expanded with increased p-values. It is noteworthy that in some cases (such as genes No. 54 and 139) not only were the genes being differentially processed but the over-all transcription level seems affected in the mutant also (Figure 7). This is not a surprise given that the pre-mRNA processing is tightly coupled with the transcription process as demonstrated by recent studies [51].

PCFS4 has been shown to regulate the APA of *FCA* pre-mRNA [13]. However, FCA could not be identified in this study. This is likely due to the sensitivity of different experimental methods and the relative abundance of the target *FCA*. Indeed, the probe intensities along the FCA gene body were hardly differentiable from those of intergenic regions around the FCA gene (Figure S1), suggesting the low abundance of FCA mRNA. It is known that the abundance of *FCA* mRNA is extremely low. In order to use Northern blot analysis to detect the *FCA* expression level, the poly(A) RNA from 400 µg of total RNA had to be loaded for a single hybridization [13]–[15]. In contrast, the cDNA target for the Arabidopsis tiling 1.0R array hybridization was prepared from 7 µg of total RNA without amplification, about 60 fold less than the amount used in Northern blot analysis (see Materials and Methods). Thus, the sensitivity of detection by RADPRE can only be limited by the tiling array technology. Under the same consideration, the DPGs and DEGs could be an underestimate of the real alternatively transcribed and processed genes.

Close examination of the probe intensities along the gene bodies for DPGs revealed that the differential processing could be explained by three simple models including alternative polyadenylation, alternative splicing and alternative transcription initiation (Figure 6; Table S2). This suggested that PCFS4 plays roles not only in polyadenylation, but also in splicing and transcription. This is not surprising given that an increasing body of evidence supports that polyadenylation is tightly coupled to the transcription and splicing processes *in vivo* [51]–[56]. Indeed, the yeast, human and *Drosophila* orthologs of Arabidopsis PCFS4 have been documented to function in transcription termination and RNA Polymerase II proccessivity by interacting with the C-terminal domain of Pol II [57]–[59]. Thus, the DPG targets of PCFS4 might open an avenue for further investigation of how polyadenylation might be coupled with other mRNA processing processes in plants.

## Materials and Methods

### Experimental design and RNA preparation

The freshly harvested *Arabidopsis thaliana* seeds of wild-type ecotype Columbia and *pcfs4-1* mutant (short as *pcfs4* in the text) [13] were germinated in 4×4 cm pots with SunGrow-360 soil (Scotts Inc.). About 100 seeds were sown for each pot, with three pots for each line in a randomized block design. The imbibed seeds were first stratified at 4 °C in the dark for two days and then grown in growth chamber at 22 °C, under 16/8 hr photoperiod. The above ground tissue of 15-day old seedlings was collected 8 hours after dawn. Total RNAs were extracted using Concert Plant RNA reagent (Invitrogen) and further treated with Turbo DNA-free (Ambion) following the manufacture's instructions. The integrity of the total RNA was examined using Agilent 2100 Bioanalyzer.

### Target preparation and hybridization

The double-stranded cDNA targets were prepared using the Affymetrix GeneChip WT Double-Stranded Target Assay kit (Affymetrix, PN900813). Briefly, first strand cDNA was synthesized from 7 µg of total RNA with random primers and Superscript II reverse transcriptase. The RNA strand was digested with RNase H and the second strand cDNA was further synthesized with DNA polymerase I. In both the 1^st^ and 2^nd^ strand synthesis, a fraction of dUTP was added in the reactions so that the uracil in the double strand cDNA could serve as a substrate of Uracil-DNA Glycosylase (UDG) for the downstream fragmentation. The fragmented double stranded cDNA was end-labeled with the biotin-labeled DLR (dual luciferase reporter) using Terminal Deoxynucleotidyl Transferase (Affymetrix, PN900812). The labeled targets were hybridized to the Arabidopsis Tiling 1.0R array (Affymetrix, PN900594) using GeneChip® Hybridization, Wash, and Stain Kit (Affymetrix, PN 900720). Hybridization was carried out on Affymetrix GeneChip Hybridization Oven 640, washed and post-hybridization stained on Affymetrix GeneChip Fluidics Station 450. Hybridization signals were collected using an Affymetrix GCS 3000 7G scanner and processed with GeneChip Operating Software. Both target preparation and hybridization were performed in the Microarray Core Facility, University of Kentucky. The final output was six CEL files containing raw data with wt1, wt2 and wt3 from wild type Col, and pcfs4.1, pcfs4.2 and pcfs4.3 from the *pcfs4* mutant. The suffix number of the three replicates denotes the block number.

### Annotation

Affymetrix provided probe information for the Arabidopsis tiling 1.0R array in the component definition file (CDF) named “At35b_MR_v04-2_TIGRv5.bpmap” (www.affymetrix.com), which denoted whether the probe was a PM (perfect match) or MM (mismatch) probe, the x and y coordinate of each probe on the chip and the exact locations of the probes in the genome. Based on “At35b_MR_v04-2_TIGRv5.bpmap” file and TAIR 8 annotation (ftp://ftp.arabidopsis.org/home/tair/Genes/TAIR8_genome_release/TAIR8_gff3/TAIR8_GFF3_genes.gff), we generated a new CDF file, named “AtTA.cdf”, in which only the probes with unique and perfect matches in the whole genome were considered. The unique and perfect match probes were picked up using MUMmer tool (http://mummer.sourceforge.net/). There were 2.8 million unique and perfect match probes in “AtTA.cdf”, accounting 87.5% of the total PM probes (3.2 million). The CDF files used in this study were essentially derived from “AtTA.cdf”.

### Data preprocessing

The raw data (CEL data) was first assessed for its quality with “image” and “box-plot” functions and then further normalized by “VSN” within the Bioconductor package *vsn* in R [35], [39], [40]. VSN is a protocol that combines the background correction and across array normalization [40]. The ratio of WT/*pcfs4* for each probe was generated using normalized data. To remove the block effects, ratios between WT and *pcfs4* were produced pair-wisely within each block, namely, wt1/pcfs4.1, wt2/pcfs4.2 and wt3/pcfs4.3. These tiling array data were deposited to GEO (GSE21250).

### Data analysis

The ratios were first log-transformed (base 2) and then the transformed ratios were grouped based on annotated exons. For each exon, whether the mean of the log-transformed ratios was equal to zero was justified based on a one-sample two-tailed T-test. As illustrated in Figure 1, any annotated transcript which had at least one exon whose mean was not equal to zero was considered to be a candidate target of PCFS4, either differentially processed or expressed. For any candidate target, a one-way ANOVA analysis and F-statistic calculation were further performed to test whether the means of its exons were equal. Any annotated transcript with equal exon means and transcripts with a single exon were considered to be a differentially expressed target of PCFS4 (DEGs), a possible indirect target of PCFS4. In contrast, any annotated transcript with unequal exon means was considered to be a differentially processed target of PCFS4, a possible direct target of PCFS4.

### Quantitative PCR

The same total RNAs mentioned above (section “Experimental design and RNA preparation”) were used to synthesize the first strand cDNA using reverse transcriptase Superscript III (Invitrogen). Two pairs of primers were designed for each candidate target, with one pair of primers to test the differentially expressed region of the transcript and the other set for the region of the transcript that showed no or less difference. The primer sequences are provided in the supplemental materials (Table S6). Real-time PCR was performed with IQ SYBR Green Supermix (170-8884, Bio-Rad) on an ICycler machine (Bio-Rad). The primer pair targeted on the beta-tublin6 transcript was used as an internal control [13]. For each primer pair, the RNAs from three WT replicates and three *pcfs4* replicates were tested. The Ct value of each primer pair was first normalized to that of beta-tublin6 followed by a two-tailed T-test for the significant difference between WT and *pcfs4*.

### Gene Ontology (GO) analysis

GOEAST is a web-based tool kit for the analysis of Gene Ontology enrichment [43]. Using the Batch Genes program within the GOEAST package, we analyzed the DPGs or the pool of DPGs and DEGs with their TAIR gene identifiers as inputs. The gene ID list of TAIR9 was used as the background gene set. The analysis was performed using the statistical method “Hypergeometric” with the significance level of FDR set at 0.01.

## Supporting Information

Figure S1 The probe intensi3es along the FCA gene and the gene At5g46490 and their surrounding intergenic regions.
(0.14 MB PDF)

Table S1 One-Sample Kolmogorov-Smirnov Test of the ratio distribution before and after log-transformation.
(0.04 MB PDF)

Table S2 The identified 142 DPGs differentially processed in the pcfs4 mutant and the possible mechanisms for differential processing.
(0.08 MB PDF)

Table S3 The identified DEG targets of PCFS4.
(0.07 MB DOC)

Table S4 The most enriched GO terms and their corresponding genes from the 68 DPG targets of PCFS4.
(0.05 MB PDF)

Table S5 The most enriched GO terms and their corresponding genes from the GO analysis of the pooled DPG and DEG targets of PCFS4.
(0.06 MB PDF)

Table S6 Sequences of the primers for the confirmation of DPG targets.
(0.03 MB PDF)
