# Supplementary material for: Ratio-Based Analysis of Differential mRNA Processing and Expression of a Polyadenylation Factor Mutant pcfs4 Using Arabidopsis Tiling Microarray
Source: PLoS One. 2011 Feb 25;6(2):e14719. doi: 10.1371/journal.pone.0014719 (PMC3045369; doi:10.1371/journal.pone.0014719)

Figure S1. The probe intensities along the FCA gene and the gene At5g46490 and their surrounding intergenic regions.

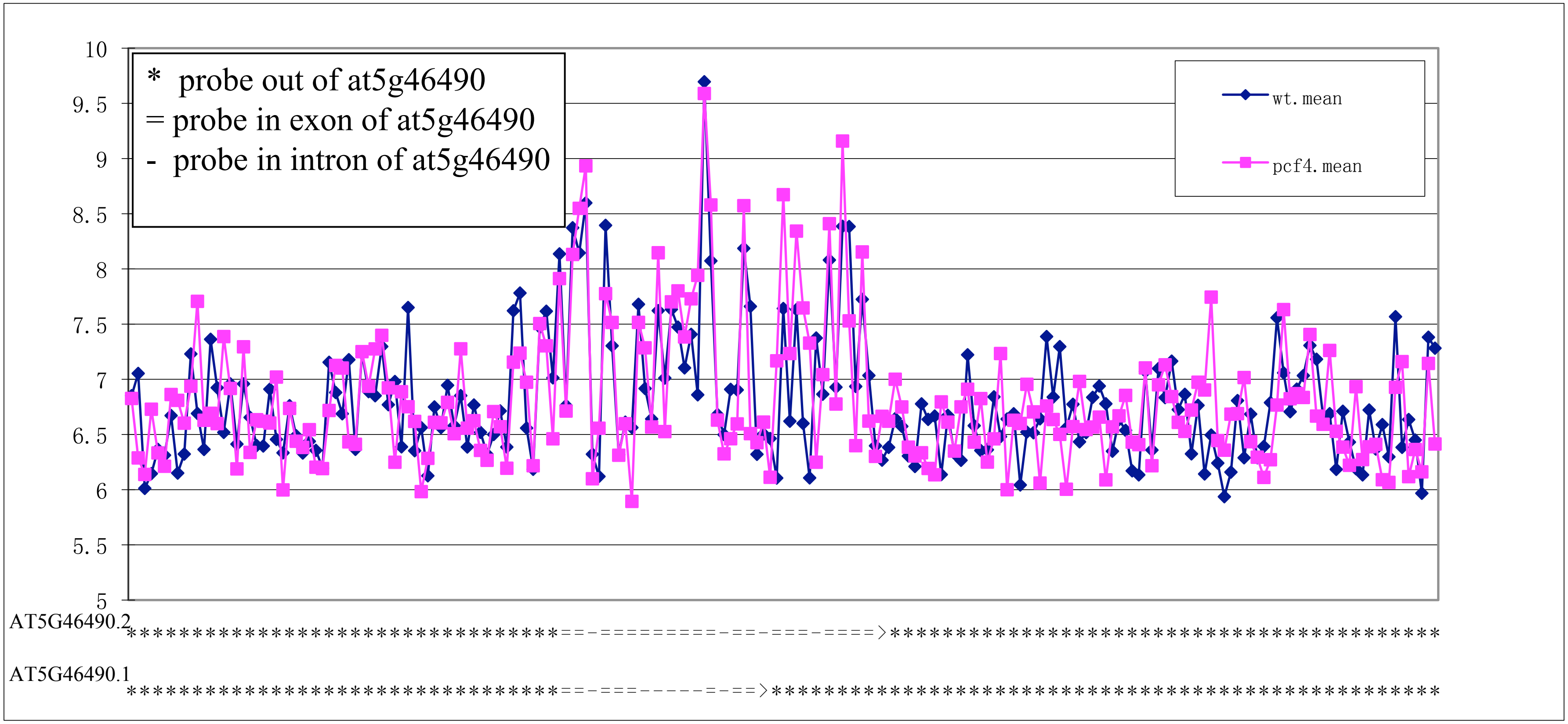

Figure S1. (Continued).

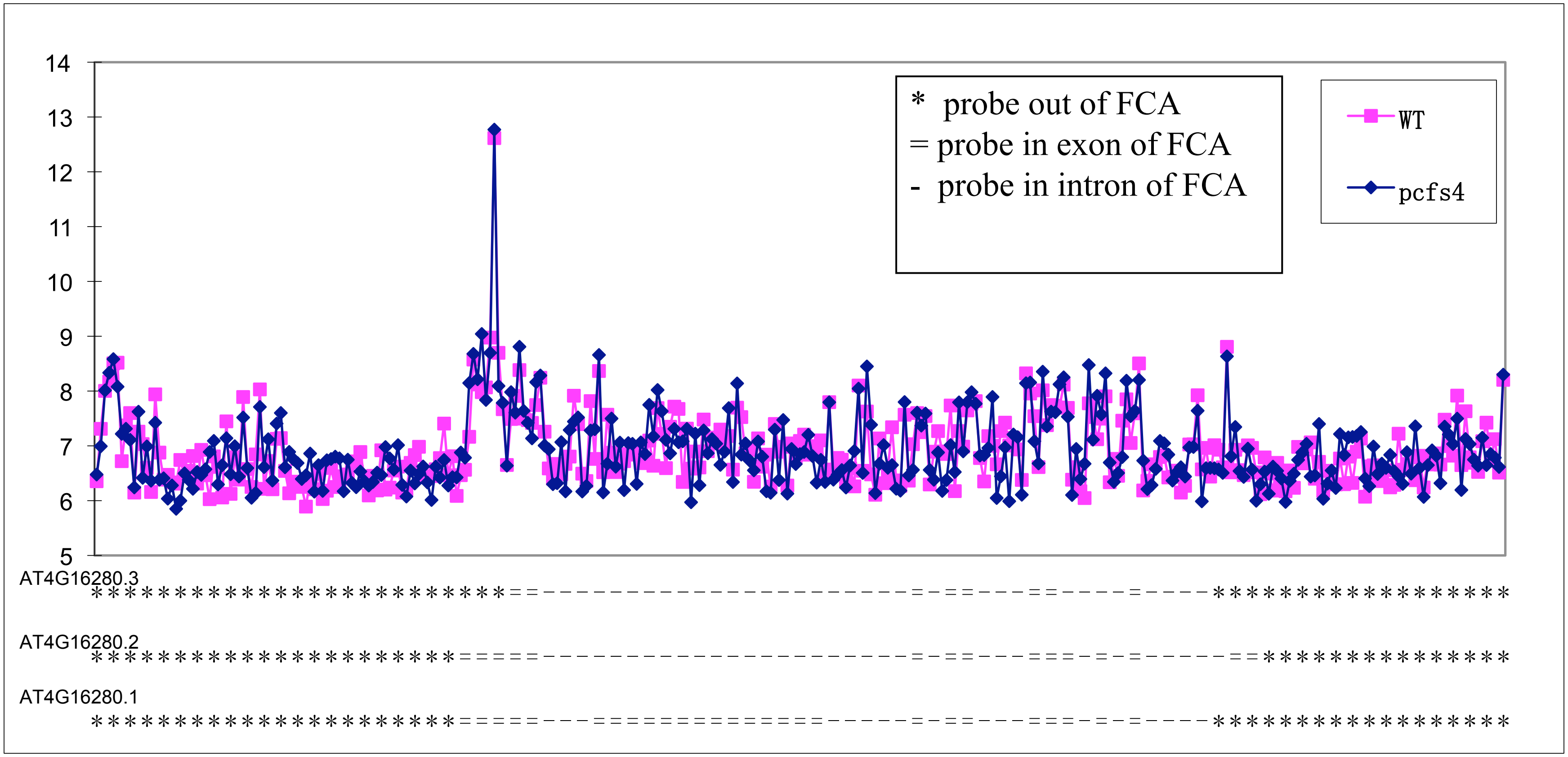

Supplement: Figure S1 — The probe intensi3es along the FCA gene and the gene At5g46490 and their surrounding intergenic regions. (0.14 MB PDF) [file pone.0014719.s001.pdf]
