# Supplementary material for: Ratio-Based Analysis of Differential mRNA Processing and Expression of a Polyadenylation Factor Mutant pcfs4 Using Arabidopsis Tiling Microarray
Source: PLoS One. 2011 Feb 25;6(2):e14719. doi: 10.1371/journal.pone.0014719 (PMC3045369; doi:10.1371/journal.pone.0014719)
Supplement: Table S1 — One-Sample Kolmogorov-Smirnov Test of the ratio distribution before and after log-transformation. (0.04 MB PDF) [file pone.0014719.s002.pdf]

Table S1. One-Sample Kolmogorov-Smirnov Test of the ratio distribution before and after log-transformation

Sample size	p-value	
	ratio	log-transformed ratio
100	0.006714	0.6267
200	0.0004489	0.8312
500	7.071e-07	0.8912
1000	2.2e-16	0.3430
5000	2.2e-16	0.06448
