# Supplementary material for: Ratio-Based Analysis of Differential mRNA Processing and Expression of a Polyadenylation Factor Mutant pcfs4 Using Arabidopsis Tiling Microarray
Source: PLoS One. 2011 Feb 25;6(2):e14719. doi: 10.1371/journal.pone.0014719 (PMC3045369; doi:10.1371/journal.pone.0014719)
Supplement: Table S2 — The identified 142 DPGs differentially processed in the pcfs4 mutant and the possible mechanisms for differential processing. (0.08 MB PDF) [file pone.0014719.s003.pdf]

Table S2. The identified 142 DPGs differentially processed in the *pcfs4* mutant and the possible mechanisms for differential processing.

Gene No.#	TAIR Gene ID	p-value*	Model**
No. 1	AT5G52910. 1	3. 39E-52	ATI
No. 2	AT5G02170. 1	1. 34E-06	APA
No. 3	AT4G38160. 2	4. 35E-06	APA
No. 4	AT4G39330. 1	4. 70E-05	APA
No. 5	AT5G62720. 2	5. 12E-05	AS
No. 6	AT5G20620. 1	9. 01E-05	APA
No. 7	AT3G26510. 1	0. 00013639	APA
No. 8	AT4G05010. 1	0. 000360295	APA
No. 9	AT3G16520. 2	0. 000387364	AS
No. 10	AT5G61560. 1	0. 000466259	AS
No. 11	AT2G22980. 2	0. 000487209	ATI
No. 12	AT2G18670. 1	0. 000579079	APA
No. 13	AT2G22980. 1	0. 000704339	ATI
No. 14	AT2G22980. 3	0. 000704339	ATI
No. 15	AT2G22980. 4	0. 000704339	ATI
No. 16	AT3G16520. 1	0. 000719896	APA
No. 17	AT2G03120. 1	0. 001114772	APA
No. 18	AT1G59870. 1	0. 001135631	AS
No. 19	AT3G26510. 3	0. 001164302	APA
No. 20	AT4G31820. 1	0. 001261359	APA
No. 21	AT5G46490. 2	0. 001381937	AS
No. 22	AT3G26510. 5	0. 001518149	ATI
No. 23	AT4G37300. 1	0. 001555258	APA
No. 24	AT5G63510. 1	0. 001650554	APA
No. 25	AT2G30770. 1	0. 002070147	ATI
No. 26	AT2G26695. 1	0. 002128813	APA
No. 27	AT3G58980. 1	0. 002301997	AS
No. 28	AT2G15890. 2	0. 002673683	APA
No. 29	AT4G36660. 1	0. 002878166	ATI
No. 30	AT3G17465. 1	0. 002934389	APA
No. 31	AT3G53130. 1	0. 002950755	AS
No. 32	AT2G28820. 1	0. 00313415	AS/APA
No. 33	AT3G02350. 1	0. 003498364	APA
No. 34	AT5G42050. 1	0. 00398813	APA
No. 35	AT5G20250. 2	0. 004152257	ATI
No. 36	AT5G18370. 1	0. 00424535	AS
No. 37	AT3G51140. 1	0. 004371274	AS

No.38	AT1G33890.1	0.004502443	AS
No.39	AT3G01270.1	0.004795879	APA
No.40	AT3G53570.2	0.004914873	AS
No.41	AT3G26510.2	0.005164614	APA
No.42	AT3G26510.4	0.005164614	APA
No.43	AT1G05520.1	0.00532054	AS
No.44	AT4G00970.1	0.005408407	APA
No.45	AT2G41800.1	0.005421864	AS
No.46	AT2G03972.2	0.005758645	APA
No.47	AT1G34350.1	0.005906869	AS
No.48	AT1G50730.1	0.006247395	APA
No.49	AT4G27060.1	0.006721021	AS
No.50	AT3G44550.1	0.006822644	APA
No.51	AT5G20250.1	0.006991082	ATI
No.52	AT4G23290.2	0.007006003	APA
No.53	AT4G23290.1	0.007320599	APA
No.54	AT4G00430.1	0.007478863	APA
No.55	AT1G70890.1	0.007627014	APA
No.56	AT2G34620.1	0.00763104	APA
No.57	AT5G44180.1	0.008602241	AS
No.58	AT3G11960.2	0.00863861	AS
No.59	AT2G32520.1	0.009035258	AS
No.60	AT3G16410.1	0.009798717	ATI
No.61	AT2G35410.1	0.010012883	APA
No.62	AT3G58020.1	0.010497984	AS/APA
No.63	AT3G61960.2	0.010545437	AS
No.64	AT4G04885.1	0.01068739	APA
No.65	AT2G19110.1	0.010893194	AS
No.66	AT4G03260.1	0.01094619	AS
No.67	AT5G45428.1	0.011199581	AS
No.68	AT3G09440.1	0.01171174	APA
No.69	AT4G34150.1	0.012036533	AS
No.70	AT1G55200.1	0.012967839	APA
No.71	AT4G22214.1	0.013555692	APA
No.72	AT2G15890.1	0.013563724	APA
No.73	AT5G65450.1	0.013677914	AS
No.74	AT3G60740.1	0.013708296	AS
No.75	AT5G22760.1	0.013730718	ATI
No.76	AT1G68590.1	0.014497027	APA
No.77	AT1G68590.2	0.014497027	APA
No.78	AT5G23000.1	0.01590454	AS

No.79	AT3G14210.1	0.01624084	APA
No.80	AT1G74690.1	0.01624197	ATI
No.81	AT5G20250.3	0.016379064	ATI
No.82	AT5G45430.1	0.016593749	AS
No.83	AT3G11964.1	0.016833029	AS
No.84	AT5G59730.2	0.016853513	AS
No.85	AT3G11960.1	0.018320281	AS
No.86	AT1G22710.1	0.018644351	APA
No.87	AT2G36830.1	0.018968756	APA
No.88	AT3G50950.2	0.019371327	APA
No.89	AT5G54280.2	0.019665961	APA
No.90	AT5G61790.1	0.019843869	AS
No.91	AT1G35710.1	0.020471311	APA
No.92	AT3G43670.1	0.021129929	AS
No.93	AT2G29630.2	0.021150113	AS
No.94	AT3G11470.1	0.021431699	ATI
No.95	AT3G11470.2	0.021431699	ATI
No.96	AT3G11470.3	0.021431699	ATI
No.97	AT3G61960.1	0.021693362	AS
No.98	AT4G14300.1	0.021925666	ATI
No.99	AT4G38040.1	0.022817521	AS
No.100	AT4G39330.2	0.022854657	APA
No.101	AT5G42900.3	0.023335569	AS
No.102	AT5G62720.1	0.024619371	APA
No.103	AT2G32870.1	0.025254017	AS
No.104	AT3G53570.4	0.025590154	AS
No.105	AT2G35640.1	0.026207558	ATI
No.106	AT2G15050.2	0.026874092	APA
No.107	AT3G30775.1	0.028143837	APA
No.108	AT4G34060.2	0.028318723	AS
No.109	AT1G27130.1	0.02954701	APA
No.110	AT1G28090.3	0.030821137	APA
No.111	AT2G41430.4	0.031222198	AS
No.112	AT3G56320.1	0.033654563	AS
No.113	AT5G35330.2	0.033924627	APA
No.114	AT1G66090.1	0.034038156	AS/APA
No.115	AT4G27310.1	0.034131075	AS
No.116	AT5G11430.1	0.035425529	AS
No.117	AT2G47850.1	0.035649781	AS
No.118	AT1G14660.1	0.036056035	APA
No.119	AT4G34060.1	0.037000839	AS

No. 120	AT1G72160.1	0.037508315	APA
No. 121	AT5G63420.1	0.038710974	AS
No. 122	AT2G22840.1	0.038836474	AS
No. 123	AT4G30470.1	0.038914326	ATI
No. 124	AT4G04460.1	0.039960955	AS
No. 125	AT1G55370.2	0.040217667	APA
No. 126	AT5G42900.2	0.040376992	AS
No. 127	AT5G48250.1	0.040929687	APA
No. 128	AT4G05170.1	0.041921161	AS
No. 129	AT4G00240.1	0.04337572	AS
No. 130	AT3G50950.1	0.045098784	APA
No. 131	AT5G65610.1	0.045230609	AS
No. 132	AT1G73870.1	0.045657614	APA
No. 133	AT1G45545.1	0.045967146	APA
No. 134	AT1G29670.1	0.046721067	APA
No. 135	AT4G10120.1	0.0470451	AS
No. 136	AT4G10120.2	0.0470451	APA
No. 137	AT3G09340.1	0.047517106	ATI
No. 138	AT3G61070.2	0.047885857	APA
No. 139	AT2G41210.1	0.048115435	APA
No. 140	AT5G38480.1	0.048664928	APA
No. 141	AT5G38480.2	0.048664928	APA
No. 142	AT2G47850.3	0.048683597	AS

Note: #The first 68 genes are defined by 1% FDR, and all 142 are defined by 2% FDR. *p-value, the p-value for the F test listed in an ascending order. **Models: ATI, alternative transcription initiation; AS, alternative splicing; APA, alternative polyadenylation.
