# Supplementary material for: Ratio-Based Analysis of Differential mRNA Processing and Expression of a Polyadenylation Factor Mutant pcfs4 Using Arabidopsis Tiling Microarray
Source: PLoS One. 2011 Feb 25;6(2):e14719. doi: 10.1371/journal.pone.0014719 (PMC3045369; doi:10.1371/journal.pone.0014719)
Supplement: Table S3 — The identified DEG targets of PCFS4. (0.07 MB DOC) [file pone.0014719.s004.pdf]

Table S3. The identified DEG targets of PCFS4

TAIR Gene ID	p-value*
**Up-regulated DEG targets of PCFS4**	
AT2G15880.1	2.02E-52
AT3G45140.1	4.88E-25
AT4G17410.1	4.10E-17
AT1G22770.1	2.47E-14
AT3G46640.2	7.47E-14
AT1G30390.1	9.63E-13
AT1G68050.1	1.24E-11
AT3G46640.1	4.41E-11
AT1G14250.1	5.87E-10
AT4G11710.1	6.57E-10
AT1G21680.1	1.18E-09
AT5G61380.1	1.31E-09
AT2G20815.1	1.49E-09
AT3G27860.1	3.61E-08
AT1G52240.1	3.95E-07
AT2G38340.1	3.98E-06
AT1G09070.1	4.57E-06
AT5G62360.1	6.75E-06
AT1G52040.1	9.08E-06
AT1G13190.1	1.02E-05
**Down-regulated DEG targets of PCFS4**	
AT1G33590.1	2.71E-19
AT2G15080.1	5.29E-18
AT5G25980.2	2.08E-17
AT2G15080.2	4.13E-17
AT5G25980.1	6.55E-17
AT5G25980.3	6.55E-17
AT2G18440.1	5.90E-16
AT4G04640.1	1.46E-15
AT3G03780.1	1.81E-15
AT3G03780.2	1.81E-15
AT3G03780.3	1.81E-15
AT1G52190.1	1.22E-14
AT2G29630.1	2.47E-14
AT1G16410.1	3.63E-14
AT3G23810.1	9.99E-14
AT1G45201.1	2.43E-13

AT1G09750.1	5.30E-13
AT1G45201.2	1.99E-12
AT4G11280.1	1.99E-12
AT4G14890.1	3.89E-12
AT4G24190.2	1.01E-11
AT4G24190.1	1.36E-11
AT4G32020.1	4.31E-11
AT4G19170.1	5.12E-11
AT2G07698.1	7.79E-11
AT5G13630.1	9.04E-11
AT5G13630.2	9.04E-11
AT1G76100.1	1.72E-10
AT1G68560.1	1.76E-10
AT1G16410.2	2.38E-10
AT5G19240.1	3.34E-10
AT3G04210.1	3.58E-10
AT1G29660.1	4.43E-10
AT4G35090.2	7.28E-10
AT5G15850.1	7.49E-10
AT3G55980.1	1.59E-09
AT4G35090.1	3.29E-09
AT3G62410.1	3.48E-09
AT1G15260.1	3.82E-09
AT3G17390.1	4.22E-09
AT5G12860.2	4.33E-09
AT3G18490.1	4.37E-09
AT5G67300.1	5.65E-09
AT3G18773.1	5.92E-09
AT2G40000.1	1.06E-08
AT1G01120.1	1.10E-08
AT1G44446.2	1.25E-08
AT1G33600.1	1.27E-08
AT1G31173.1	1.53E-08
AT4G24570.1	2.30E-08
AT5G37770.1	2.51E-08
AT2G10410.1	2.87E-08
AT4G34980.1	3.14E-08
AT3G52500.1	3.14E-08
AT5G12860.1	3.74E-08
AT1G02150.1	3.75E-08
AT1G26150.1	3.99E-08

AT1G72150.1	4.41E-08
AT2G15050.1	5.97E-08
AT4G03260.2	6.00E-08
AT2G31880.1	6.68E-08
AT5G15845.1	7.26E-08
AT1G20510.2	7.28E-08
AT1G20510.1	8.01E-08
AT3G47250.1	9.13E-08
AT5G26200.1	9.62E-08
AT1G78850.1	1.58E-07
AT4G29780.1	1.81E-07
AT5G61420.2	2.66E-07
AT5G13770.1	2.76E-07
AT1G79245.1	3.18E-07
AT3G18060.1	3.75E-07
AT3G47250.2	4.73E-07
AT4G30440.1	4.84E-07
AT2G40140.2	6.17E-07
AT3G47250.3	6.73E-07
AT2G07734.1	1.13E-06
AT2G40140.1	1.25E-06
AT5G66520.1	1.50E-06
AT2G15050.3	1.68E-06
AT5G36120.1	2.29E-06
AT4G36648.1	2.44E-06
AT2G46220.1	2.66E-06
AT1G60950.1	2.80E-06
AT5G61420.1	2.93E-06
AT2G29510.1	3.98E-06
AT2G22500.1	4.57E-06
AT4G19100.1	5.59E-06
AT1G78820.1	6.93E-06
AT2G28790.1	7.55E-06
AT3G61820.1	9.37E-06
AT1G79720.1	1.68E-05
AT3G15730.1	8.63E-05
AT2G42690.1	0.000151212

Note: *, denote the p-value based on T-statistic test.
