# Supplementary material for: Ratio-Based Analysis of Differential mRNA Processing and Expression of a Polyadenylation Factor Mutant pcfs4 Using Arabidopsis Tiling Microarray
Source: PLoS One. 2011 Feb 25;6(2):e14719. doi: 10.1371/journal.pone.0014719 (PMC3045369; doi:10.1371/journal.pone.0014719)
Supplement: Table S4 — The most enriched GO terms and their corresponding genes from the 68 DPG targets of PCFS4. (0.05 MB PDF) [file pone.0014719.s005.pdf]

Table S4. The most enriched GO terms and their corresponding genes from the 68 DPG targets of PCFS4

Gene ID	Gene Symbol
**Response to stress; GO:0006950; p=1.39E-05**	
AT3G45140	LOX2
AT1G33590	
AT2G15080	AtRLP19
AT4G11280	ACS6
AT3G04210	
AT5G67300	MYBR1
AT2G40000	HSPRO2
AT5G37770	TCH2
AT1G52040	MBP1
AT2G15890	MEE14
AT5G20250	DIN10
AT4G00430	PIP1;4
AT4G34150	
AT3G14210	ESM1
AT3G30775	ERD5
**Regulation of flower development; GO:0009909; p=7.78E-05**	
AT1G68050	FKF1
AT5G15850	COL1
AT5G37770	TCH2
AT4G04885	PCFS4
