# Supplementary material for: Ratio-Based Analysis of Differential mRNA Processing and Expression of a Polyadenylation Factor Mutant pcfs4 Using Arabidopsis Tiling Microarray
Source: PLoS One. 2011 Feb 25;6(2):e14719. doi: 10.1371/journal.pone.0014719 (PMC3045369; doi:10.1371/journal.pone.0014719)
Supplement: Table S5 — The most enriched GO terms and their corresponding genes from the GO analysis of the pooled DPG and DEG targets of PCFS4. (0.06 MB PDF) [file pone.0014719.s006.pdf]

Table S5. The most enriched GO terms and their corresponding genes from the GO analysis of the pooled DPG and DEG targets of PCFS4

TAIR Gene ID	Gene Symbol
**Sulfur metabolic process; GO:0006790; p=6.11E-09**	
AT1G59870	PEN3
AT2G30770	CYP71A13
AT3G61440	CYSC1
AT3G16410	NSP4
AT3G14210	ESM1
AT2G29630	THIC
AT5G25980	TGG2
AT3G03780	ATMS2
AT1G16410	CYP79F1
AT4G11280	ACS6
AT3G17390	MTO3
**Response to stress; GO:0006950; p=9.56E-09**	
AT1G59870	PEN3
AT5G46490	AT5G46490.1
AT2G30770	CYP71A13
AT2G15890	MEE14
AT5G20250	DIN10
AT5G18370	AT5G18370.1
AT4G36140	AT4G36140.1
AT3G61440	CYSC1
AT4G00430	PIP1;4
AT3G09440	AT3G09440.1
AT4G34150	AT4G34150
AT3G14210	ESM1
AT3G50950	ZAR1
AT3G30775	ERD5
AT2G41430	ERD15
AT1G66090	AT1G66090.1
AT3G45140	LOX2
AT1G33590	AT1G33590
AT2G15080	AtRLP19
AT1G22770	GI
AT4G11280	ACS6
AT3G04210	AT3G04210.1
AT4G35090	CAT2
AT5G67300	MYBR1
AT3G17390	MTO3
AT2G40000	HSPRO2
AT1G01120	KCS1
AT5G37770	TCH2

AT1G20510	OPCL1
AT5G61420	MYB28
AT2G40140	CZF1
AT1G52040	MBP1
**Circadian rhythm; GO:0007623; p=4.15E-06**	
AT1G22770	GI
AT3G46640	PCL1
AT1G68050	FKF1
AT5G61380	TOC1
AT5G15850	COL1
**S-glycoside metabolic process; GO:0016143; p=7.18E-06**	
AT1G59870	PEN3
AT3G16410	NSP4
AT3G14210	ESM1
AT5G25980	TGG2
AT1G16410	CYP79F1
**Regulation of flower development; GO:0009909; p=4.00E-04**	
AT4G31820	ENP
AT4G04885	PCFS4
AT1G68050	FKF1
AT5G15850	COL1
AT5G37770	TCH2
