# Supplementary material for: Ratio-Based Analysis of Differential mRNA Processing and Expression of a Polyadenylation Factor Mutant pcfs4 Using Arabidopsis Tiling Microarray
Source: PLoS One. 2011 Feb 25;6(2):e14719. doi: 10.1371/journal.pone.0014719 (PMC3045369; doi:10.1371/journal.pone.0014719)
Supplement: Table S6 — Sequences of the primers for the confirmation of DPG targets. (0.03 MB PDF) [file pone.0014719.s007.pdf]

Table S6: Sequences of the primers for the confirmation of DPG targets

Gene No.	Gene ID	Primer Name	Primer Sequence
No.1	At5g52910	p1024	CGCTGATGTGAACGTCTGTT
		p1025	CAAGCAGCAAACAGGATCAA
		p921	TGCCTCCCAATAAACCAGAG
		p922	CATCACTGCCGCTAGACAGA
No.2	At5g02170	p1026	CACACGTTCGATGAGGAAGA
		p1027	TTGCTGTTTGCTTGGTGAAG
		p943	CGGCTCTGATTGGATCTTTC
		p944	CCGCTTACAATGCCGATTAT
No.22	At3g26510	p1275	TCATCAACCACCACGTCATC
		p1276	CAGTGATTGCCGTTGTGAAC
		p1277	CCGGAGATTTGACTTTGGAA
		p1278	AACGGTTGGACGAATAAATCC
No.23	At4g37300	p1271	TGGACAGAGACGACGATGAG
		p1272	GGATGGTCGGTGATTTTGTC
		p1273	GGAGACAACAAACAAGTTGCAG
		p1274	CTAGAAGGTCTGCTGGTTGG
No.31	At3g53130	p1279	ACTGCAAAACCTACGCGTCT
		p1280	TGGGATTTCGATGAATTGGTC
		p1281	AAGGCAATGTCTGGCGTATC
		p1282	AATCCCCTGCACTACCTCCT
No.35	At5g20250	p1283	GGCGTCACAGAGTTGCTACA
		p1284	AGATGAGCGAATACGAAGCTG
		p1285	TTCCGGTTTAAGCTCTGGTG
		p1286	CCGTTAGCTCCGTCAGACTC
No.54	AT4G00430	p1287	GTCTGTTCTTGGCTCGGAAG
		p1288	TGGAAACCTTTGACGACTCC
		p1289	TGGCAAAGGGTCTCTGAAGT
		p1290	ACCAACCCGAGAACTTGATG
No.69	AT4G34150	p1295	ATGATGGCGGGTATACAAGG
		p1296	GCAGGTTCTGGTACGGTGAC
		p1297	ATACTCAGCACCGCCTTCTG
		p1298	GAGGGTATCCTGATGGCTGA
No.72	AT2G15890	p1299	CGTTCGTTTCATCTCCCAAT
		p1300	TCTCTGGTGCTTGAACAACG
		p1301	TCTATTCTTGATGATGTTTGTTCTCC
		p1302	CATCTTTGCATCACCAAACAA
No.79	AT3G14210	p1303	TTGGTGAGTGTTCTGGGAGTC
		p1304	TAGGGCCAATAGGTTTGTGG
		p1305	AATACAAAACCGGCAACGAG
		p1306	GAACGGCGTTGTAGAAATCG
No.87	AT2G36830	p1307	CGTTGGCTGAGTTCATTTCA
		p1308	CCAATGAAAGCACCGAAAGT
		p1309	TCTTGTGTAATTTTATTCGACTTTGA
		p1310	GAAATTGCAATGGCCACTCT
No.101	AT5G42900	p1311	GGCACGGTGGTAATTCTCAT

		p1312	GAGCCAGAGCTGCATATTCC
		p1313	TGAGAGGTCAGATGGTGAAGC
		p1314	ATGAATCCACTCACATGAGCA
No.106	AT2G15050	p1319	CGGTCCAATCACAGCAAAG
		p1320	CCGACGGTCTAGGGTTGTAA
		p1321	AGTTAACTGGACATAAATCGGAAAG
		p1322	ACAAAAATTCAACCACAAGACCA
No.107	AT3G30775	p1315	CACGATTCAGGCGTACTTGA
		p1316	AAACCAGAACCGTTTGATGC
		p1317	CGGGATTGCGTAAAGAGAGA
		p1318	CATGAAAAATATTTAGCCACAGGA
No.115	AT4G27310	p1323	CTTACGCCGTGGAAAGCTAC
		p1324	ACCGTCACCGTGATCTTCTC
		p1325	CGATCGGCGTTTAAATCAAC
		p1326	CAGAAAACTGAACGGTGGTG
No.125	AT1G55370	p1327	TTGCTTCTTTGCCACTTCAG
		p1328	ATCTTCGAAAGTGGCTCCAT
		p1329	TCTGGTTGTGTATGGAGAGGTG
		p1330	CGGTTAGATAAGGGGGTTGC
No.139	AT2G41210	p1331	GGGTTCCTACATTGCCTTCA
		p1332	CCTTCAGCGTCCAATCTCAT
		p1333	CTCAAGTTGTCTCTGGCGTTT
		p1334	TCATTGCTATGGCTTTTAGCTG
